# Sustainable and recyclable heterogenous palladium catalysts from rice husk-derived biosilicates for Suzuki-Miyaura cross-couplings, aerobic oxidations and stereoselective cascade carbocyclizations

**DOI:** 10.1038/s41598-020-63083-8

**Published:** 2020-04-14

**Authors:** Samson Afewerki, Ana Franco, Alina M. Balu, Cheuk-Wai Tai, Rafael Luque, Armando Córdova

**Affiliations:** 1 Department of Natural Sciences, Mid Sweden University, Sundsvall, SE-85170 Sweden; 2 Berzelii Center EXSELENT on Porous Materials, Stockholm University, Stockholm, SE-10691 Sweden; 3 Departamento de Química Orgánica, Universidad de Córdoba, Campus de Rabanales Edificion Marie Curie (C-3), Ctra Nnal IV-A, Km 396, 14014 Cordoba, Spain; 4 Department of Environmental and Materials Chemistry, Stockholm University, Stockholm, SE-10691 Sweden; 5 Peoples friendship University of Russia (RUDN University), 6 Miklukho Maklaya str, 117198 Moscow, Russia

**Keywords:** Heterogeneous catalysis, Porous materials

## Abstract

A new eco-friendly approach for the preparation of sustainable heterogeneous palladium catalysts from rice husk-derived biogenic silica (RH_P_-Si and RH_U_-Si). The designed heterogeneously supported palladium species (RH_P_-Si-NH_2_-Pd and RH_U_-Si-NH_2_-Pd) were fully characterized and successfully employed as catalysts for various chemical transformations (C–C bond-forming reactions, aerobic oxidations and carbocyclizations). Suzuki-Miyaura transformations were highly efficient in a green solvent system (H_2_O:EtOH (1:1) with excellent recyclability, providing the cross-coupling products with a wide range of functionalities in high isolated yields (up to 99%). Palladium species (Pd(0)-nanoparticles or Pd(II)) were also efficient catalysts in the green aerobic oxidation of an allylic alcohol and a co-catalytic stereoselective cascade carbocyclization transformation. In the latter case, a quaternary stereocenter was formed with excellent stereoselectivity (up to 27:1 dr).

## Introduction

**Figure 1:**
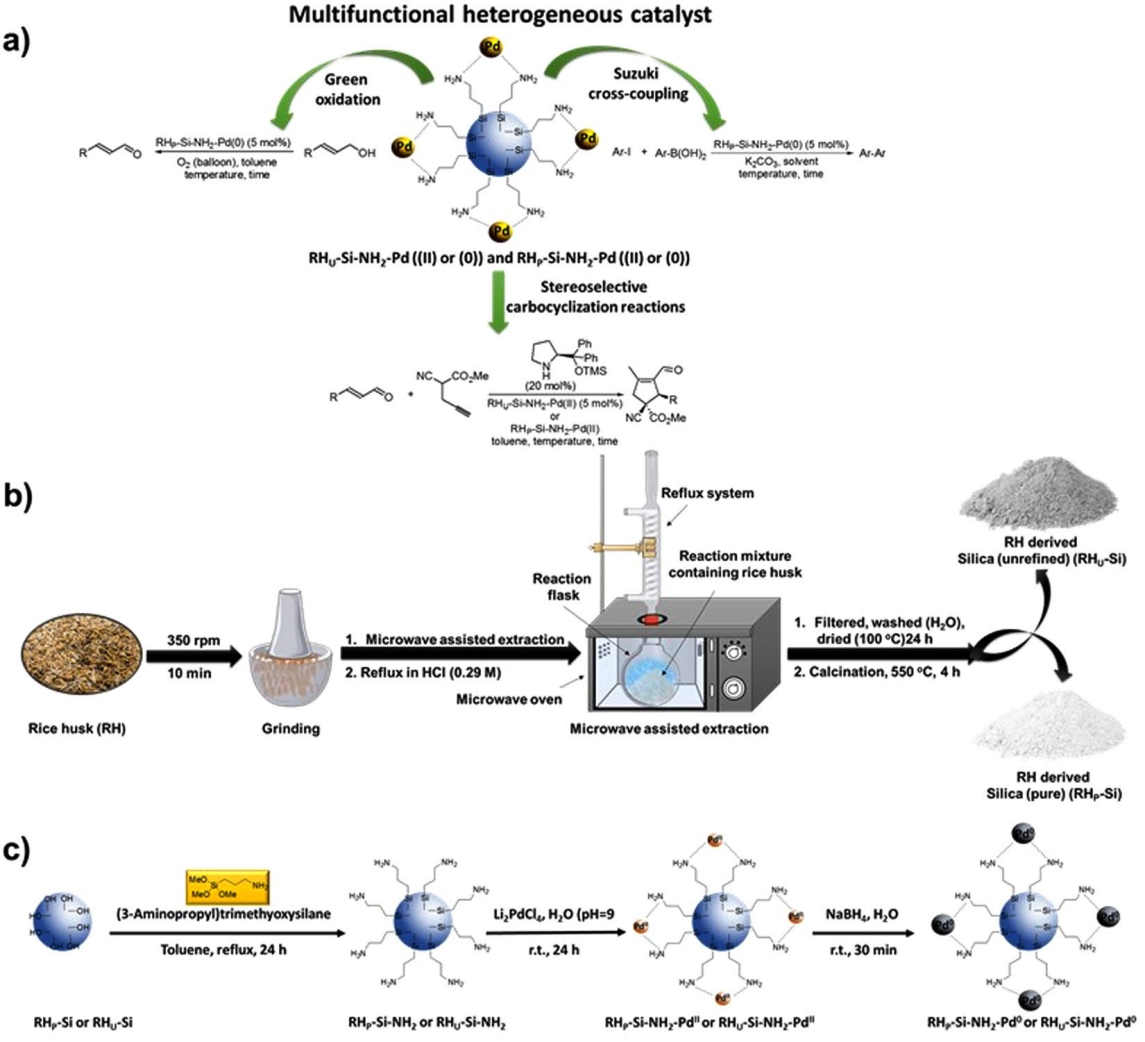
(**a**) Catalytic applications of the versatile Pd heterogeneous catalyst into various green chemical transformation. (**b**) Novel, low-cost and energy-efficient environmentally friendly approach for the preparation of rice husk (RH) based silica (RH_U_-Si and RH_P_-Si) through a simple process involving grinding, microwave assisted extraction, washing and calcination. (**c**) Synthetic strategy for further modification of the RH-derived silica (RH_U_-Si and RH_P_-Si) into a palladium based heterogenous catalyst ((RH_P_-Si-NH_2_-Pd(II), RH_U_-Si-NH_2_-Pd(II), RH_P_-Si-NH_2_-Pd(0) and RH_U_-Si-NH_2_-Pd(0)).

Rice husk (RH) is a major waste product from the rice industry with high content of silica. This abundant material is a sustainable and cheap raw material and therefore within the context of sustainability^1^. De facto, rice husk ash (RHA) is known to contain 94% silica^2^. Here, silica is a very important component for various industrial and biomedical applications^3^, being high surface area silicates highly desirable and good candidates as catalyst support^4^. For these reasons the development of efficient methods for the preparation of silica and silica based materials are of high interest. In general, the production of silica from RH is energy intensive i.e. obtained by burning RH in a muffle furnace at high temperature (500, 600 or 700 °C)^5,6^. Nevertheless, even though there are some reports on simple and energy-efficient method for the generation of silica from RH, drawbacks such as risk for mineral contamination^7^, several acid and based extraction steps or tedious approaches^8^ are encountered^9^. In this context, novel methods for the extraction of silica in an eco-friendly, cost-efficient, scalable and facile approach can be highly attractive. This report discloses the preparation of palladium based multifunctional heterogenous catalysts from RH-derived silica as support. There are reports on the immobilization of various metals on RH and their use as heterogeneous catalysts for chemical syntheses^10–16^. In this context, Chang and co-workers developed [Pd(NH_3_)_4_]^2+^-modified nanopore silica, derived from RH and further demonstrated their use in the solvent-free Suzuki-Miyaura cross-coupling reaction^17^. However, some examples of the coupling reactions resulted in very poor reactivity (~4% yield). Within this theme, Gogoi and co-workers also developed a highly efficient RH based Pd(II)-Schiff base complex heterogenous catalyst for the Suzuki- Miyaura coupling reaction in water^18^. Even though the catalyst was recyclable and reusable up to 6 cycles, it successively lost its activity (1^st^ cycle 98% and 6^th^ cycle 90% yield). The same reaction was employed by Boruah et al. by using recyclable Pd(OAc)_2_ in neat Water Extract of Rice Straw Ash (WERSA), nevertheless, moderate to high yields (45–90% yields) and low enduring recyclability was observed (1^st^ cycle 88% and 6^th^ cycle 65% yield)^19^. Additionally, Liu et al. demonstrated the preparation of porous silica derived from acid leached RH after calcination as support for palladium and cerium (IV) oxide (CeO_2_). The catalyst was employed for the catalytic methane combustions^20^. Moreover, Esmaeilpour et al. disclosed the preparation of dendrimer-encapsulated Pd(0) nanoparticles immobilized on nanosilica (nSiO2-dendrimer-Pd(0)) and their application in the Sonogashira-Hagihara reactions in the absence of any copper and phosphorous ligand in water under aerobic conditions^21^. Despite these notable advances, there is a need to find novel, facile and green approaches to obtain high purity and surface area silica rice husk (RH-Si) for further catalyst design into multifunctional, recyclable and highly efficient heterogenous catalytic systems. Heterogenous catalysts offers several advantages such as allowing the facile and practical recycling of the catalyst, reuse in several cycles without any loss of efficiency and avoiding the leaching of expensive and toxic metals^22^. All of these features characterize a sustainable and eco-friendly technology. Based on the above challenges and our previous experience in developing palladium based heterogenous catalysts for various green chemical transformations^23–29^, we designed an eco-friendly approach for the preparation of heterogeneous and multifunctional palladium catalysts suitable for a wide spectrum of chemical transformations. The activity and versatility of the designed catalysts was demonstrated in the Suzuki-Miyaura cross-coupling reaction, aerobic oxidation approach of allylic alcohols and stereoselective cascade carbocyclization reactions (Fig. 1a)^30^.

## Results and Discussion

**Figure 2:**
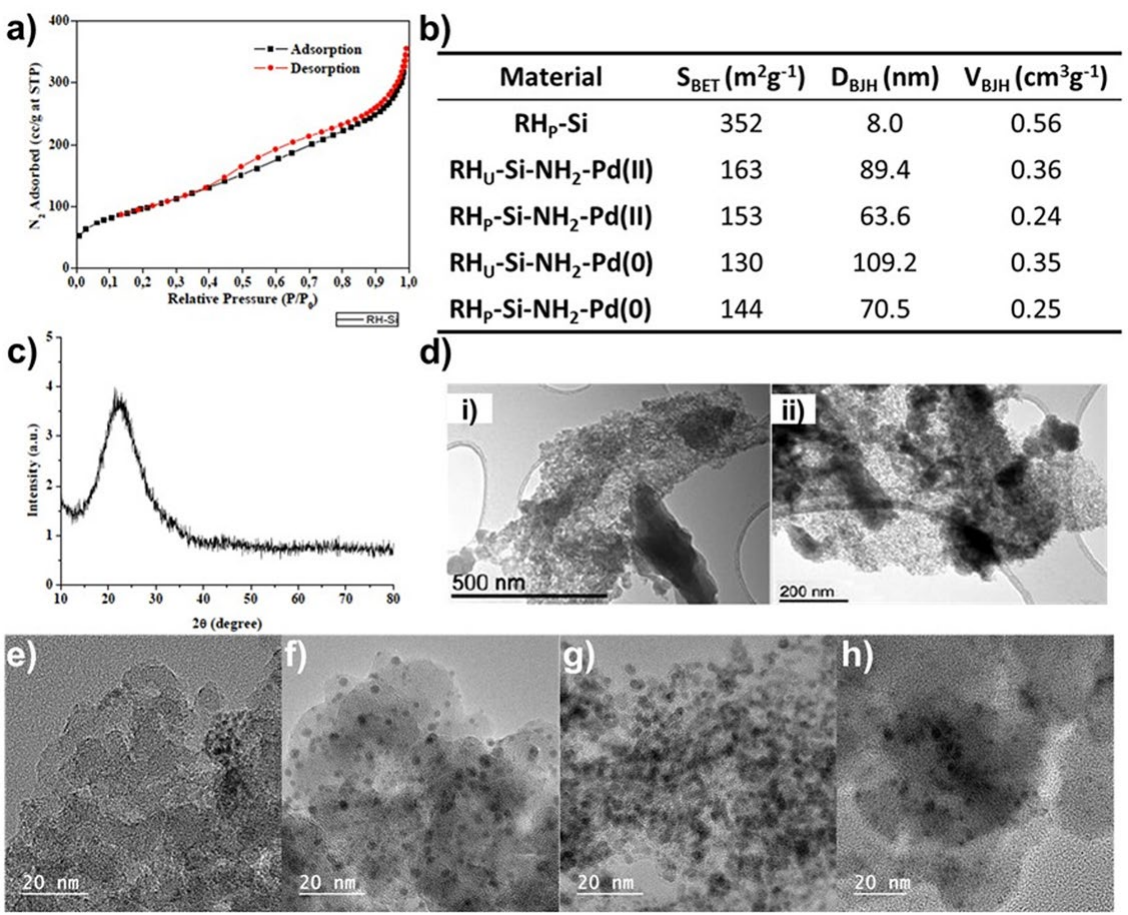
(**a**) N sorption isotherms of the silica materials derived from rice husk (RH_P_-Si). (**b**) Textural properties of RH_P_-Si and corresponding palladium based heterogenous catalysts (RH_P_-Si-NH_2_-Pd(II), RH_U_-Si-NH_2_-Pd(II), RH_P_-Si-NH_2_-Pd(0) and RH_U_-Si-NH_2_-Pd(0)). Definitions: S_BET_: specific surface area calculated by the Brunauer-Emmett-Teller (BET) equation. D_BJH_: mean pore size diameter calculated by the Barret-Joyner-Halenda (BJH) equation. V_BJH_: pore volumes calculated by the Barret-Joyner-Halenda (BJH) equation. (**c**) Powder X-ray diffraction (XRD) patterns of RH_P_-Si. (**d**) Transmission Electron Microscopy (TEM) of RH_P_-Si at different magnifications, at (i) 500 nm and (ii) 200 nm. TEM of the various palladium heterogenous catalysts (**e**) RH_U_-Si-NH_2_-Pd(II) (f) RH_U_-Si-NH_2_-Pd(0) (**g**) RH_P_-Si-NH_2_-Pd(II) (**h**) RH_P_-Si-NH_2_-Pd(0).

Initially, RH derived silica was obtained through a previously reported facile and novel approach (Fig. 1b)^31^. RH was grinded, underwent microwave-assisted extraction and then further purification (washing and calcination) to afford the silica products. Two type of silica materials were prepared, one unrefined biosilicate (RH_U_-Si, 90% purity, grey color) and the second one, pure biosilicate (RH_P_-Si, >98% purity, white color). Subsequently, the homogenous palladium catalyst was incorporated onto the RH_U_-Si and RH_P_-Si via conjugation with amino groups through a silylation step (RH_U_-Si-NH_2_ and RH_p_-Si-NH_2_), followed by treatment with the palladium precursor (Li_2_PdCl_4_) providing the heterogenous palladium catalysts (RH_U_-Si-NH_2_-Pd(II) and RH_p_-Si-NH_2_-Pd(II)). These two heterogenous palladium (II) catalysts were simply converted to the corresponding RH_U_-Si-NH_2_-Pd(0) and RH_p_-Si-NH_2_-Pd(0) by a reduction step (Fig. 1c). The fabricated materials were thoroughly characterized (Fig. 2). Firstly, the porosity and pore size of the RH based materials (RH_P_-Si, RH_U_-Si-NH_2_-Pd(II)/Pd(0) and RH_P_-Si-NH_2_-Pd(II)/Pd(0)) were determined by nitrogen physisorption experiments (Figs. 2a and S3−10). Unmodified RH_P_-Si displayed a Brunauer-Emmet-Teller (BET) with a surface area of ca. 352 m^2^/g^−1^ and with mesoporous characteristics (8.0 nm) and pore volume of 0.56 cm^3^/g^−1^ (Fig. 2b). However, both surface area and pore volume decreased after palladium incorporation, whilst the pore size distribution changed to macropores (e.g. RH_U_-Si-NH_2_-Pd(II) = 163 m^2^/g^−1^, 89.4 nm and 0.36 cm^3^/g^−1^). Such is a normal behaviour observed in our previous reports, indicating the binding and incorporation of palladium into the material^23,27,32^. The elemental analysis confirmed the palladium content of the various heterogenous catalysts as 20.30 wt% for RH_U_-Si-NH_2_-Pd(II), RH_P_-Si-NH_2_-Pd(II) = 19.11 wt%, RH_U_-Si-NH_2_-Pd(0) = 19.05 wt% and RH_P_-Si-NH_2_-Pd(0) = 16.90 wt%, respectively.

Moreover, the surface area decreased and pore size increased when the Pd(II) was reduced to Pd(0) (e.g. RH_P_-Si-NH_2_-Pd(II) = 153 m^2^/g^−1^ and 63.6 nm; RH_P_-Si-NH_2_-Pd(0) = 144 m^2^/g^−1^ and 70.5 nm). X-ray diffraction (XRD) patterns of the RH_P_-Si displayed a strong broad peak at about 22° 2θ angle indicating its amorphous structure (Fig. 2c). This is also consistent with previous reports on amorphous silica derived from rice husk^33^. The amorphous characteristics of the RH_P_-Si could also be confirmed by Scanning electron microscopy (SEM) micrographs (Figure S11). Additionally, Transmission electron microscopy (TEM) of the RH_P_-Si at different magnification are presented in Fig. 2d, which further demonstrated the amorphous and porous structure. After palladium incorporation, a clear difference could be observed in the TEM, where well dispersed and spherical palladium nanoparticles could be visualized (Fig. 2e–h).

**Figure 3:**
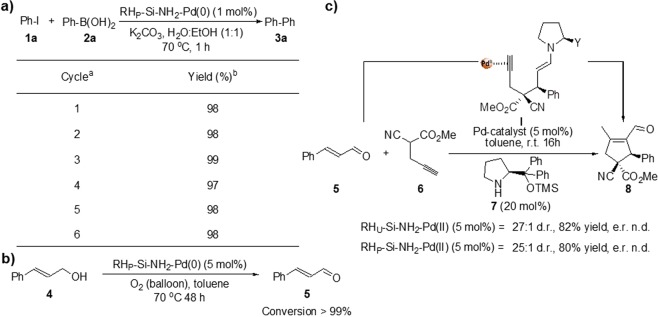
(**a**) Table demonstrating the recyclability study of RH_P_-Si-NH_2_-Pd(0) catalyst in the catalytic Suzuki-Miyaura reaction. (**b**) Expanding the reaction scope of the devised catalyst RH_P_-Si-NH_2_-Pd(0) for the oxidation of the cinnamyl alcohol **4** to the corresponding cinnamic aldehyde **5**. (**c**) Expanding the reaction scope for the combined heterogeneous palladium and amine catalyzed stereoselective carbocylization reaction between the cinnamic aldehyde **5** and propargylcyanomalonate **6**. Definitions: n.d; not determined, d.r.; diastereomeric ratio and e.r.; enantiomeric ratio.

Upon characterization, the catalytic performance of the designed heterogenous palladium catalysts were further evaluated. The Suzuki cross-coupling reaction was selected as model reaction. The initial reaction between iodobenzene **1a** and phenylboronic acid **2a**, in the presence of potassium carbonate (K_2_CO_3_)^29^ and catalytic amounts of RH_U_-Si-NH_2_-Pd(II) (5 mol%) in water (H_2_O) provided the corresponding diphenyl product **3a** in 54% isolated yield after 3 h at 100 °C (Table 1, entry 1). Further screening of the solvents showed that dimethylformamide (DMF) displayed the best efficiency among the investigated (toluene (85%) and ethanol (EtOH) (95%)) to afford **3a** in 98% yield (Table 1, entries 2–4). However, enduring our vision in designing an eco-friendly process, we decided to mix H_2_O and ethanol (EtOH) (1:1) as reaction medium, and to our delight, the reaction provided the product **3a** in 99% yield (Table 1, entry 5). This improvement could be due to the improved solubility of the substrates with the addition of ethanol than having solely H_2_O as solvent. It is well-known that silicate is not soluble or show very low solubility in water^34^. No differences in catalytic activity between the various palladium catalysts were observed (Table 1, entries 5–8), and moreover, a decrease in catalyst amount (from 5.0 to 0.25 mol%) did not negatively impact the reaction efficiency (Table 1, entries 8–11). However, when the amount of the Pd-catalyst was decreased to 0.10 mol% the efficiency was decreased and provided the product **3a** with 81% yield (Table 1, entry 12). Enduring the fine-tuning of the reaction, a decrease in reaction temperature and time were further investigated, where 1 h reaction time at 70 °C worked well (Table 1, entries 13–15). However, further decrease of the temperature to 50 °C decreased the efficiency of the reaction (72% yield, Table 1, entry 16). Delighted by these findings, the substrate scope of the reaction was further explored, which showed that the reaction tolerated a wide range of functionalities with both aryl iodide **1a** and aryl bromide **1b** and various boronic acids **2** (Table 2). Nevertheless, a slight decrease in yields was observed for the reaction between bromobenzene **1b** and phenylboronic acid **2a** and between the iodobenzene **1a** and 4-(trifluoromethyl) phenylboronic acid **2e** providing products **3a** and **3 f** (85 and 88% yields, Table 2, entries 2 and 8). Overall, the coupling reaction showed high efficiency and provided the coupling products in high yields (up to 99%). Since the recyclability of a heterogenous catalyst is an eminence feature both in the economic and environmental aspects, the recyclability and reusability of the devised palladium heterogenous catalysts were further studied. The heterogenous systems could be recycled for 6 consecutive cycles without losing any efficiency (Fig. 3a). Notable all the four heterogenous palladium catalysts (RH_U_-Si-NH_2_-Pd(II), RH_P_-Si-NH_2_-Pd(II only), RH_U_-Si-NH_2_-Pd(0), RH_P_-Si-NH_2_-Pd(0) were recycled at least one cycle. However RH_P_-Si-NH_2_-Pd(0) catalyst was selected for further recycling study. Moreover, no leaching was observed as determined by elemental analysis performed on the filtrate after the hot filtration and after the completion of the reaction.

**Table 1:** Reaction optimization conditions of the Suzuki cross-coupling reaction.


Entry^a^	Pd-catalyst (mol%)	Solvent	Yield (%)^b^
1	RH_U_-Si-NH_2_-Pd(II) (5)	H_2_O	54
2	RH_U_-Si-NH_2_-Pd(II) (5)	toluene	85
3	RH_U_-Si-NH_2_-Pd(II) (5)	EtOH	95
4	RH_U_-Si-NH_2_-Pd(II) (5)	DMF	98
5	RH_U_-Si-NH_2_-Pd(II) (5)	H_2_O:EtOH (1:1)	99
6	RH_P_-Si-NH_2_-Pd(II) (5)	H_2_O:EtOH (1:1)	98
7	RH_U_-Si-NH_2_-Pd(0) (5)	H_2_O:EtOH (1:1)	98
8	RH_P_-Si-NH_2_-Pd(0) (5)	H_2_O:EtOH (1:1)	99
9	RH_P_-Si-NH_2_-Pd(0) (1)	H_2_O:EtOH (1:1)	98
10	RH_P_-Si-NH_2_-Pd(0) (0.5)	H_2_O:EtOH (1:1)	98
11	RH_P_-Si-NH_2_-Pd(0) (0.25)	H_2_O:EtOH (1:1)	99
12	RH_P_-Si-NH_2_-Pd(0) (0.10)	H_2_O:EtOH (1:1)	81
13^c^	RH_P_-Si-NH_2_-Pd(0) (0.25)	H_2_O:EtOH (1:1)	98
14^d^	RH_P_-Si-NH_2_-Pd(0) (0.25)	H_2_O:EtOH (1:1)	98
**15**^**e**^	**RH**_**P**_**-Si-NH**_**2**_**-Pd(0) (0.25)**	**H**_**2**_**O:EtOH (1:1)**	**98**
16 ^f^	RH_P_-Si-NH_2_-Pd(0) (0.25)	H_2_O:EtOH (1:1)	72

^[a]^Reaction conditions: Pd-catalyst (mol%), iodobenzene **1a** (204.0, 1.0 mmol, 1.0 equiv.), phenylboronic acid **2a** (146.3 mg, 1.2 mmol, 1.2 equiv.), K_2_CO_3_ (414.6 mg, 3.0 mmol, 3.0 equiv.), solvent (3 mL), 100 °C, 3 h. ^[b]^Yield of purified product **3a** after silica chromatography. ^[c]^The reaction was run for 1 h. ^[d]^The reaction was performed at 70 °C. ^[e]^The reaction was run for 1 h at 70 °C. ^[f]^The reaction was run for 1 h at 50 °C.

**Table 2:** Scope of the Suzuki coupling reaction catalyzed by RH_P_-Si-NH_2_-Pd(0).


Entry^a^	Ar^1^-X	Ar^2^-B(OH)_2_	Product **3**	Yield (%)^b^
1	**1a**		**3a**	98
2	**1b**		**3a**	85
3	**1a**		**3b**	95
4	**1a**		**3c**	99
5	**1b**		**3c**	92
6	**1c**		**3d**	98
7	**1a**		**3e**	94
8	**1a**		**3f**	88
9	**1d**		**3g**	99
10	**1c**		**3h**	99

^[a]^Reaction conditions: RH_P_-Si-NH_2_-Pd(0) (1.6 mg, 0.25 mol%), aryl iodine **1** (1.0 mmol, 1.0 equiv.), arylboronic acid **2** (1.2 mmol, 1.2 equiv.), K_2_CO_3_ (414.6 mg, 3.0 mmol, 3.0 equiv.), H_2_O:EtOH (1:1, 3 mL), 70 °C, 1 h. ^[b]^Yield of purified product **3** after silica chromatography.

To further broaden the application of the synthesized Pd catalyst, the selected optimum system (RH_P_-Si-NH_2_-Pd(0) was employed in the aerobic oxidation of cinnamyl alcohol **4** to the corresponding aldehyde as model reaction^32^. The reaction proceeded efficiently to afford cinnamic aldehyde **5** (>99%) as only product after 48 h, in toluene at 70 °C, employing 5 mol% of the catalyst in the presence of oxygen gas (Fig. 3b). In addition to the cross-coupling and oxidation reactions, the reaction portfolio was further expanded for the application of the heterogenous palladium catalyst in amine/palladium co-catalyzed carbocyclization reactions (Fig. 3c). The reaction was conducted between cinnamic aldehyde **5** and propargylcyanomalonate **6** in the presence of catalytic amount of palladium heterogenous catalyst (RH_U_-Si-NH_2_-Pd(II) and RH_P_-Si-NH_2_-Pd(II) (5 mol%)) and the chiral amine catalyst **7** (20 mol%). The chemical transformation proceeds via formation of the enaminyne **I** intermediate^35^, and subsequent stereoselectivity nucleophilic enamine addition provided the carbocycle **8**, in high yields (up to 82%) and diastereoselectivities (up to 27:1 dr determined through ^1^H-NMR analysis and the e.r. where not determined. However, the obtained optical rotation ([α]_D_^25^ = −6.31 (c = 1.0 CHCl_3_)) resembles to the previously reported ee of >97.5:2.5 er) (Fig. 3c)^23^. These all examples highlight the versatility and simplicity of the devised RH-based heterogenous palladium catalyst employed in various relevant chemical transformations.

## Conclusion

A highly efficient Pd-heterogeneous catalyst from the biomass-based Rice husk waste was synthesized. The novel preparation approach integrating the valorization of renewable starting materials represents a green, facile and simple eco-friendly method for catalyst design. The devised heterogenous palladium catalysts were proved to be highly versatile, being successfully employed in Suzuki-Miyaura cross-couplings (high product yields, up to 99%, wide range of functionalities), the aerobic oxidation of cinnamoyl alcohol and the amine catalyzed stereoselective carbocylization reaction (cyclopentene derivatives obtained in high yields and stereoselectivities, up to 82%, 27:2 dr). Additionally, the Pd system was highly recyclable and could be reused after simple centrifugation in 6 cycles without any loss of efficiency. The disclosed protocol represents a green and sustainable chemical approach that may find relevant and suitable future applications in additional chemical transformations.

## Methods

### General and materials

Chemicals and solvents were either purchased *puriss p. a*. from commercial suppliers or were purified by standard techniques. Commercial reagents were used as purchased without any further purification. Aluminum sheet silica gel plates (Fluka 60 F254) were used for thin-layer chromatography (TLC), and the compounds were visualized by irradiation with UV light (254 nm) or by treatment with a solution of phosphomolybdic acid (25 g), Ce(SO_4_)_2_·H_2_O (10 g), conc. H_2_SO_4_ (60 mL), and H_2_O (940 mL), followed by heating. ^1^H NMR spectra were recorded on a Bruker Avance (500 MHz or 400 MHz) spectrometer. Chemical shifts are reported in ppm from tetramethylsilane with the solvent resonance resulting from incomplete deuterium incorporation as the internal standard (CDCl_3_: δ 7.26 ppm). Data are reported as follows: chemical shift, multiplicity (s = singlet, d = doublet, q = quartet, br = broad, m = multiplet), and coupling constants (Hz), integration. ^13^C NMR spectra were recorded on a Bruker Avance (125.8 MHz or 100 MHz) spectrometer with complete proton decoupling. Chemical shifts are reported in ppm from tetramethylsilane with the solvent resonance as the internal standard (CDCl_3_: δ 77.16 ppm). Gas sorption measurements were carried out on a Micrometrics ASAP2020 analyzer and recorded at 77 K. N_2_ adsorption measurements on the RH-Si were performed at 77 K by using a Micromeritics ASAP 2000 volumetric adsorption analyzer. The samples were degassed for 24 h at 130 °C under vacuum (P_o_ < 10^−2^ Pa) and subsequently analyzed. Surface area of the RH-Si was calculated according to the Brunauer-Emmet-Teller (BET) equation. Mean pore size diameter and pore volumes were obtained from porosimetry data by using Barret-Joyner-Halenda (BJH) method. Wide-angle X-ray diffraction experiments were recorded on a Pan-Analytic/Philips X’pert MRD diffractometer (40 kV, 30 mA) with CuK_α_ (λ = 0.15418 nm) radiation. Scans were performed over a 2θ = 10–80 °C at step size of 0.0188 with a counting time per step of 5 s. TEM image of the RH-Si was obtained on JEM 2010F (JEOL) and Phillips Analytical FEI Tecnai 30 microscopes. All other TEM experiments were carried out on a 200 kV JEOL JEM-2100F field-emission electron microscope equipped with an ultra-high-resolution pole piece. A Gatan ultra-high tilt tomography holder was used. TEM samples were prepared by crushing, and the tomography data was acquired between −60° and +60° with 1° increments. Each image per tilt angle was recorded with a Gatan Ultrascan 1000 camera. The data acquisition was assisted by a commercial tomography packed, TEMography (version 2.15.07) developed by JEOL System Technology Co. Ltd. SEM micrographs of the RH-Si was recorded in a JEOL-SEM JSM-6610 LV scanning electron microscope in backscattered electron mode at 3/15 kV. Elemental analyses were carried out by Medac LTD Analytical and chemical consultancy services (United Kingdom) by ICP-OES.

### Preparation of the RH_P_-Si-Pd-heterogeneous catalysts Rice husk silica preparation (RH_P_-Si)

The particle size of the rice husk (RH) was reduced by grinding in a Retsch-PM-100 planetary ball mill using a 125 mL reaction chamber end eighteen stainless steel balls (10 nm diameter, 5 g weight). Milling was conducted at 350 rpm for 10 min. The obtained silica from RH was treated to microwave assisted extraction in ETHOS-ONE. The RH was then refluxed in a 0.29 M HCl solution at 300 W for 30 min. The silica solution was cooled to room temperature, filtered and washed with distilled water and then dried in oven at 100 °C for 24 h. The resulting solid was calcined in a furnace at 550 °C for 4 h to obtain pure silica (RH_P_-Si).

### General preparation of the RH_P_-Si-NH_2_

The preparation of the RH_P_-Si-NH_2_-Pd-catalyst started with the amino functionalization of the RH_P_-Si. Dry toluene (20 mL) was added to the RH_P_-Si (1.38 g, 22.2 mmol mg, 1.0 equiv.), followed by addition of a solution of 3-aminopropyltrimethoxy silane (7.8 mL, 44.4 mmol, 2.0 equiv.) in toluene (10 mL). The mixture was stirred under nitrogen for 10 minutes, and then refluxed for 24 h. The mixture was allowed to cool to room temperature and the solid was collected by filtration and washed several times with toluene, ethanol, acetone and dichloromethane to remove any unreacted precursor. The material was further dried under vacuum giving RH_P_-Si-NH_2_ (1.405 g).

### General preparation of the RH_P_-Si-NH_2_-Pd(II)

The amino-functionalized RH_P_-Si-NH_2_ (1.0 g) was suspended in deionized water (15 mL) and the solution was pH-adjusted to pH 9, by the use of 0.1 N LiOH. In parallel Li_2_PdCl_4_ (600 mg) was diluted in deionized water (10 mL) and the solution was pH-adjusted to pH 9, by the use of 0.1 N LiOH. This solution was transferred to the flask containing RH_P_-Si-NH_2_ solution. The reaction was stirred at room temperature for 24 h. Subsequently, the suspension was then centrifuged, and the solid material was further washed with water (3 × 40 mL) and acetone (3 × 40 mL) and dried overnight under vacuum to afford RH_P_-Si-NH_2_-Pd(II) (1.408 g). Elemental analysis on the Pd content were 20.30 wt.% for RH_U_-Si-NH_2_-Pd(II) and 19.11 wt.% for RH_P_-Si-NH_2_-Pd(II).

### General preparation of the RH_P_-Si-NH_2_-Pd(0)

RH_P_-Si-NH_2_-Pd(II) (500 mg) was suspended in deionized water (15 mL), followed by slow addition of a solution of NaBH_4_ (310.2 mg, 8.2 mmol) in water (5 mL) at room temperature. The reaction was stirred at room temperature for 30 minutes. Afterwards the solution was centrifuged and the solid diluted with water (3×40 mL), acetone (3×40 mL) and centrifuged. The material was then dried overnight under vacuum providing RH_P_-Si-NH_2_-Pd(0). Elemental analysis on Pd content were 19.05 wt.% for the RH_U_-Si-NH_2_-Pd(0) and 16.90 wt.% for RH_P_-Si-NH_2_-Pd(0), respectively.

### General procedure for Pd-catalyst catalyzed Suzuki-Miyaura reaction (Table 1)

A microwave vial equipped with a magnetic stir bar was charged with the Pd-catalyst (mol%), phenylboronic acid **2a** (146.4 mg, 1.2 mmol, 1.2 equiv.), K_2_CO_3_ (414.6 mg, 3.0 mmol, 3.0 equiv.), followed by addition of solvent (3.0 mL). Subsequently, iodobenzene **1a** (204.0 mg, 1.0 mmol, 1.0 equiv.) was added and the reaction mixture heated and run for the temperature and time stated at Table 1. Next, the reaction mixture was either centrifuged and the solid diluted with acetone (3×10 mL) and centrifuged and then concentrated before purification or directly subjected to flash chromatography on silica (petroleum ether/EtOAc 100−90%) affording the pure product **3a**.

### Procedure for RH_P_-Si-NH_2_-Pd (0) catalyst catalyzed Suzuki-Miyaura reaction (Table 2)

A microwave vial equipped with a magnetic stir bar was charged with RH_P_-Si-NH_2_-Pd(0) catalyst (1.6 mg, 0.0025 mmol, 0.25 mol%), arylboronic acid **2** (1.2 mmol, 1.2 equiv.), K_2_CO_3_ (414.6 mg, 3.0 mmol, 3.0 equiv.), followed by addition of H_2_O:EtOH (1:1, 3.0 mL). Subsequently, aryl halide **1** (1.0 mmol, 1.0 equiv.) was added and the reaction mixture heated to 70 °C and stirred for 1 h. Next, the reaction mixture was either centrifuged and the solid diluted with acetone (3 × 10 mL) and centrifuged and then concentrated before purification or directly subjected to flash chromatography on silica (petroleum ether/EtOAc, 100–90%) affording the pure products **3**.

### Procedure for the recycling of RH_P_-Si-NH_2_-Pd(0) catalyst

A microwave vial equipped with a magnetic stir bar was charged with RH_P_-Si-NH_2_-Pd(0) catalyst (6.3 mg, 0.01 mmol, 1.0 mol%), phenylboronic acid **2a** (146.4 mg, 1.2 mmol, 1.2 equiv.), K_2_CO_3_ (414.6 mg, 3.0 mmol, 3.0 equiv.), followed by addition of H_2_O:EtOH (1:1, 3.0 mL). Subsequently, iodobenzene **1a** (204.0 mg, 1.0 mmol, 1.0 equiv.) was added and the reaction mixture heated to 70 °C and stirred for 1 h. Next, the reaction mixture was centrifuged and the solid diluted with acetone (3×10 mL) and centrifuged. The collected liquid was concentrated and purified by flash chromatography on silica (petroleum ether/EtOAc 100−90%) affording the pure product **3a**. The solid catalyst was diluted with water (10 mL) in order to remove remaining base and centrifuged. The solid catalyst was further diluted with acetone (2 × 10 mL) and centrifuged. Afterwards the solid heterogeneous catalyst was dried under vacuum and then further used in next cycle. Notable all the four heterogenous palladium catalysts (RH_U_-Si-NH_2_-Pd(II), RH_U_-Si-NH_2_-Pd(0), RH_P_-Si-NH_2_-Pd(II), RH_P_-Si-NH_2_-Pd(0)) were recycled at least one cycle. RH_P_-Si-NH_2_-P(0) was selected for further cycle studies.

### Typical procedure for the hot-filtration test

A microwave vial equipped with a magnetic stir bar was charged with pure RH_P_-Si-NH_2_-Pd(0) catalyst (1.6 mg, 0.25 mol%), phenylboronic acid **2a** (146.4 mg, 1.2 mmol, 1.2 equiv.), K_2_CO_3_ (414.6 mg, 3.0 mmol, 3.0 equiv.), followed by addition of solvent (3.0 mL). Subsequently, iodobenzene **1a** (204.0 mg, 1.0 mmol, 1.0 equiv.) was added and the reaction mixture heated to 70 °C. RH_P_-Si-NH_2_-Pd(0) catalyst was removed through centrifugation after 30% conversion was reached and the solid free filtrate was allowed to stir for 24 h reaction conditions. Analysis of the reaction mixture showed that no further conversion of the substrate had occurred.

### Procedure for the catalytic aerobic oxidation

To a suspension of RH_P_-Si-NH_2_-Pd(0) (5 mol% Pd to **4**, 7.6 mg) in toluene (0.5 mL) placed in an oven-dried microwave vial equipped with a magnetic stir bar was charged with cinnamyl alcohol **4** (32.3 mg, 0.24 mmol, 1.2 equiv.). The vial was capped, evacuated and an O_2_-balloon was connected to the reaction vessel. The reaction mixture was stirred at 70 °C for 48 h affording the the corresponding cinnamic aldehyde **5**.

### General procedure for the combined transition metal/amine catalytic reaction using propargylcyanomalonate

An oven-dried microwave vial equipped with a magnetic stir bar was charged with propargylcyanomalonate **6** (16.1 mg, 0.12 mmol, 1.2 equiv.) and Pd-catalyst (5 mol%, 0.005 mmol), followed by addition of toluene (0.100 mL) and the resulting mixture was stirred at room temperature for 5 min. In parallel to the above procedure, an oven- dried vial was charged with the cinnamic aldehyde **5** (13 mg, 0.1 mmol, 1.0 equiv.), aminocatalyst **7** (6.5 mg, 0.02 mmol, 20 mol%) and followed by addition of toluene (0.15 mL), after stirring at room temperature for additional 5 min, the resulting mixture was transferred to the vial containing the mixture of palladium catalyst and propargylcyanomalonate *via* a syringe. (Total volume of Toluene = 0.25 mL, Final concentration = 0.8 M to aldehyde). The reaction was stirred for 24 h at room temperature. The conversions and diastereomeric ratio were monitored by ^1^H NMR analysis of the crude mixture. Upon completion, the mixture was directly subjected to flash chromatography on silica (pentane/EtOAc) affording the pure products in **8**.

## Supplementary information

Supporting information.
