# Supplementary material for: Sustainable and recyclable heterogenous palladium catalysts from rice husk-derived biosilicates for Suzuki-Miyaura cross-couplings, aerobic oxidations and stereoselective cascade carbocyclizations
Source: Sci Rep. 2020 Apr 14;10:6407. doi: 10.1038/s41598-020-63083-8 (PMC7156381; doi:10.1038/s41598-020-63083-8)
Supplement: Supplementary file 1 — Supporting information. [file 41598_2020_63083_MOESM1_ESM.doc]

# Supplementary Information

**Sustainable and recyclable heterogenous palladium catalysts from rice husk-derived biosilicates for Suzuki-Miyaura cross-couplings, aerobic oxidations and stereoselective cascade carbocyclizations**

Samson Afewerki,^*,†,‡^ Ana Franco,^┴^ Alina M. Balu,^*,┴^ Cheuk-Wai Tai,^†,¶^ Rafael Luque,^┴, ¥^ and Armando Córdova^*,†,‡^

‡Department of Natural Sciences, Mid Sweden University, Sundsvall SE-85170, Sweden.

†Berzelii Center EXSELENT on Porous Materials, Stockholm University, Stockholm, SE-10691, Sweden.

┴Departamento de Química Orgánica, Universidad de Córdoba, Campus de Rabanales Edificiion Marie Curie (C-3), Ctra Nnal IV-A, Km 396, 14014, Cordoba, Spain.

¶Department of Environmental and Materials Chemistry, Stockholm University, Stockholm, SE-10691, Sweden.

¥Peoples friendship University of Russia (RUDN Univeristy), 6 Miklukho Maklaya str., 117198, Moscow, Russia.

**Contents**

Characterization

Spectra of 1-Phenylnaphthalene

- Figure S1. The ^1^H-NMR spectrum of the 1-Phenylnaphthalene

- Figure S2. The ^13^C-NMR spectrum of the 1-Phenylnaphthalene

Characterization of the various silica-based materials

- Figure S3. N_2_ sorption isotherms of the RH_U_-Si-NH_2_-Pd(II)

- Figure S4. Pore size distribution of the RH_U_-Si-NH_2_-Pd(II) using the adsorption

isotherms with density functional theory

- Figure S5. N_2_ sorption isotherms of the RH_U_-Si-NH_2_-Pd(0)

- Figure S6. Pore size distribution of the RH_U_-Si-NH_2_-Pd(0) using the adsorption

isotherms with density functional theory

- Figure S7. N_2_ sorption isotherms of the RH_P_-Si-NH_2_-Pd(II)

- Figure S8. Pore size distribution of the RH_P_-Si-NH_2_-Pd(II) using the adsorption

isotherms with density functional theory

- Figure S9. N_2_ sorption isotherms of the RH_P_-Si-NH_2_-Pd(0)

- Figure S10. Pore size distribution of the RH_P_-Si-NH_2_-Pd(0) using the adsorption

isotherms with density functional theory

- Figure S11. The SEM image of the RH-Si

**Characterization**

**1,1’-biphenyl**[[1]](#footnote-2): White solid. **^1^H NMR (400MHz, CDCl_3_):** δ 7.64 (d, *J* = 8.1 Hz, 4H), 7.48 (t, *J* = 7.5 Hz, 4H), 7.64 (t, *J* = 7.5 Hz, 2H); **^13^C NMR (100MHz, CDCl_3_):** 141.4, 128.9, 127.4, 127.3.

**4-methyl-1,1’-biphenyl**^1^: White solid. **^1^H NMR (400MHz, CDCl_3_):** δ 7.67 (d, *J* = 7.8 Hz, 2H), 7.59 (d, *J* = 8.2 Hz, 2H), 7.51 (t, *J* = 7.6 Hz, 2H), 7.41 (t, *J* = 7.3 Hz, 1H), 7.34 (d, *J* = 7.8 Hz, 2H), 2.48 (s, 3H); **^13^C NMR (100MHz, CDCl_3_):** 141.3, 138.5, 137.1, 129.6, 128.8, 127.1, 127.1, 21.2.

**1-Phenylnaphthalene**[[2]](#footnote-3): White solid. **^1^H NMR (400MHz, CDCl_3_):** δ 7.79 (q, *J* = 15.8, 8.9 Hz, 2H), 7.73 (d, *J* = 8.2 Hz, 1H), 7.42-7.26 (m, 9H); **^13^C NMR (100MHz, CDCl_3_):** 140.8, 140.3, 133.9, 131.7, 130.1, 128.3, 127.7, 127.3, 127.0, 126.1, 125.8, 125.4.

**(*E*)-3-styrylpyridine**[[3]](#footnote-4): White solid. **^1^H NMR (400MHz, CDCl_3_):** δ 8.59 (d, *J* = 2.0 Hz, 1H), 8.36 (dd, *J* = 4.8, 1.4 Hz, 1H), 7.66 (dt, *J* = 8.0, 3.8 Hz, 1H), 7.38 (d, *J* = 8.1 Hz, 2H), 7.24 (t, *J* = 7.4 Hz, 2H), 7.19-7.14 (m, 1H), 7.12 (dd, *J* = 8.0, 4.8 Hz, 1H), 7.01 (d, *J* = 16.4 Hz, 1H), 6.91 (d, *J* = 16.4 Hz, 1H); **^13^C NMR (100MHz, CDCl_3_):** 148.5, 148.4, 136.6, 133.0, 132.6, 130.8, 128.8, 128.2, 126.7, 124.8, 123.5.

**(*E*)-Stilbene**[[4]](#footnote-5): White solid. **^1^H NMR (400MHz, CDCl_3_):** δ 7.54 (d, *J* = 8.3 Hz, 4H), 7.39 (t, *J* = 7.5 Hz, 4H), 7.31-7.25 (m, 2H), 7.14 (s, 2H); **^13^C NMR (100MHz, CDCl_3_):** 137.5, 128.8, 127.8, 126.7.

**4-(trifluoromethyl)-1,1’-biphenyl**^1^: White solid. **^1^H NMR (400MHz, CDCl_3_):** δ 7.72 (s, 4H), 7.62 (d, J = 8.0 Hz, 2H), 7.50 (t, *J* = 7.6 Hz, 2H), 7.43 (tt, *J* = 8.2, 1.3 Hz, 1H); **^13^C NMR (100MHz, CDCl_3_):** 144.9, 139.9, 129.1, 128.3, 127.6, 127.4, 125.9, 125.8.

**3-Methoxybiphenyl(ref)**[[5]](#footnote-6): White solid. **^1^H NMR (400MHz, CDCl_3_):** δ 7.46 (d, *J* = 7.4 Hz, 2H), 7.30 (t, *J* = 14.4 Hz, 2H), 7.26-7.17 (m, 2H), 7.11-6.98 (m, 2H), 6.76 (d, *J* = 8.4 Hz, 1H), 3.71 (s, 3H); **^13^C NMR (100MHz, CDCl_3_):** 159.9, 142.8, 141.1, 129.7, 128.7, 127.4, 127.2, 119.7, 112.9, 112.7, 55.3.

**3-(*p*-tolyl)pyridine**[[6]](#footnote-7): Yellow oil. **^1^H NMR (400MHz, CDCl_3_):** δ 8.83 (d, *J* = 2.0 Hz, 1H), 8.55 (dd, *J* = 4.8, 1.5 Hz, 1H), 7.82 (tt, *J* = 8.0, 4.0 Hz, 1H), 7.46 (d, *J* = 8.1 Hz, 2H), 7.31 (dd, *J* = 8.1, 4.9 Hz, 1H), 7.26 (d, *J* = 8.1 Hz, 2H), 2.39 (s, 3H); **^13^C NMR (100MHz, CDCl_3_):** 148.1, 138.0, 136.6, 134.9, 134.1, 129.8, 126.9, 123.5, 21.1.

**Methyl 1-cyano-3-formyl-4-methyl-2-phenylcyclopent-3-enecarboxylate**: oil. **^1^H NMR (400MHz, CDCl_3_):** δ 9.92 (s, 1H), 7.38-7.32 (m, 3H), 7.17-7.15 (m, 2H), 4.72 (bs, 1H), 3.89 (s, 3H), 3.41 (d, *J* = 14.8 Hz, 1H), 3.26 (dt, *J* = 12.4 Hz, *J´*= 1.2 Hz, 1H), 2.33 (d, *J* = 0.8 Hz, 3H); **^13^C NMR (100MHz, CDCl_3_):** 186.2, 168.8, 157.8, 136.8, 136.6, 129.1, 128.7, 128.0, 117.4, 58.4, 54.4, 51.7, 47.9, 14.3. [α]_D_^25^ = −6.31 (c = 1.0 CHCl_3_)

**Spectra of 1-Phenylnaphthalene**

**Figure S1.** The ^1^H-NMR spectrum the 1-Phenylnaphthalene

**Figure S2.** The ^13^C-NMR spectrum the 1-Phenylnaphthalene

**Characterization of the various silica-based materials**


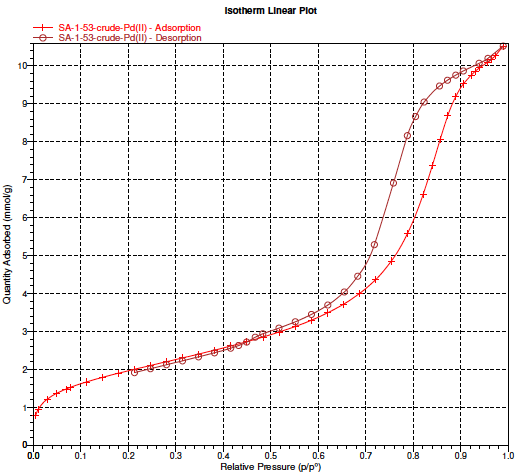


**Figure S3.** N_2_ sorption isotherms of the RH_U_-Si-NH_2_-Pd(II)


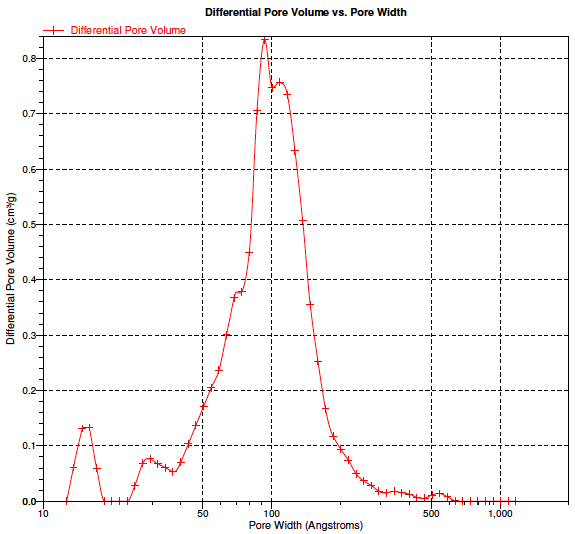


**Figure S4.** Pore size distribution of the RH_U_-Si-NH_2_-Pd(II) using the adsorption isotherms with density functional theory

**
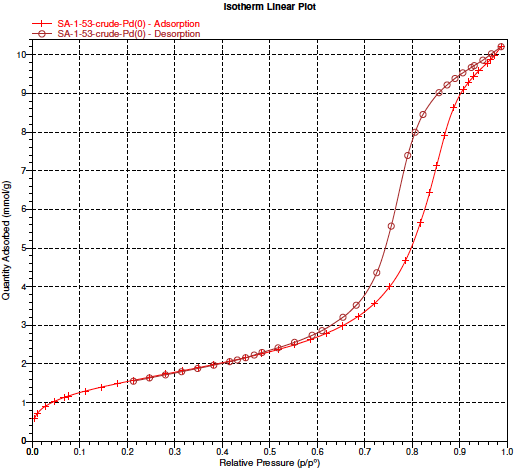
**

**Figure S5.** N_2_ sorption isotherms of the RH_U_-Si-NH_2_-Pd(0)

**
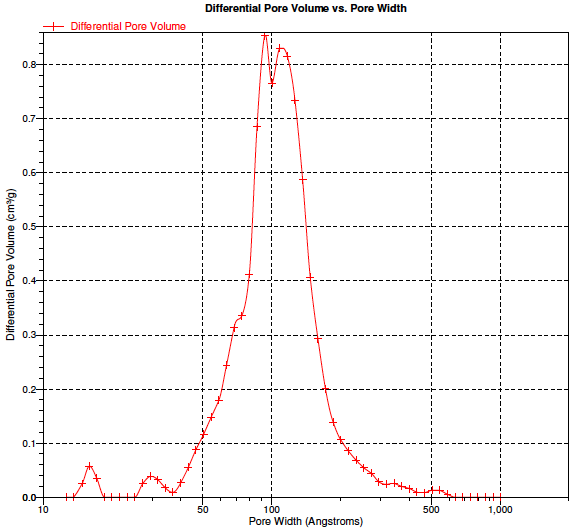
**

**Figure S6.** Pore size distribution of the RH_U_-Si-NH_2_-Pd(0) using the adsorption isotherms with density functional theory


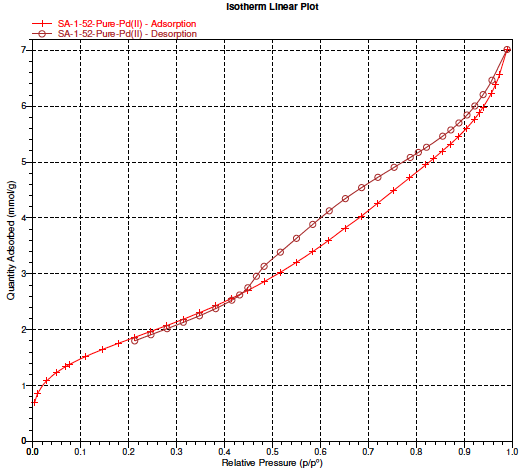


**Figure S7.** N_2_ sorption isotherms of the RH_P_-Si-NH_2_-Pd(II)


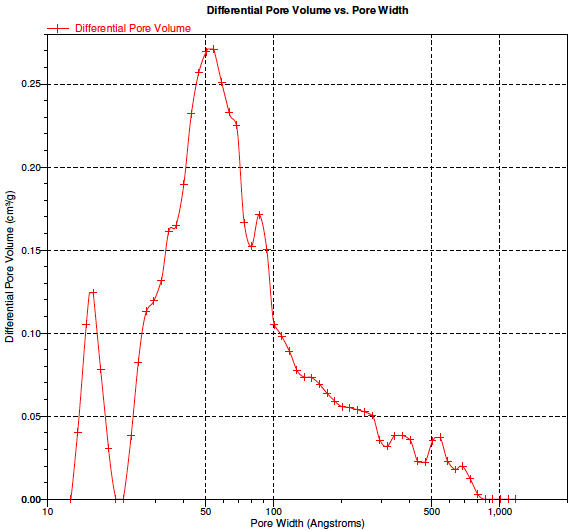


**Figure S8.** Pore size distribution of the RH_P_-Si-NH_2_-Pd(II) using the adsorption isotherms with density functional theory

**
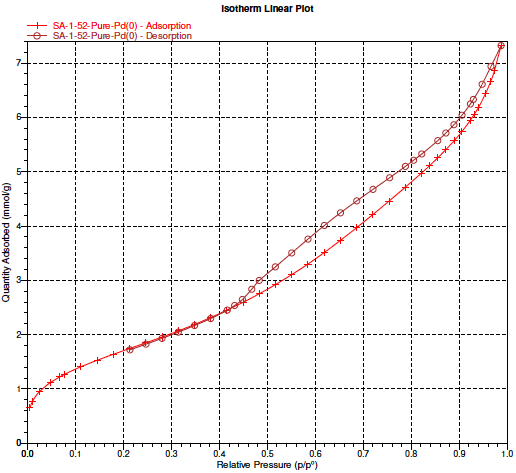
**

**Figure S9.** N_2_ sorption isotherms of the RH_P_-Si-NH_2_-Pd(0)

**
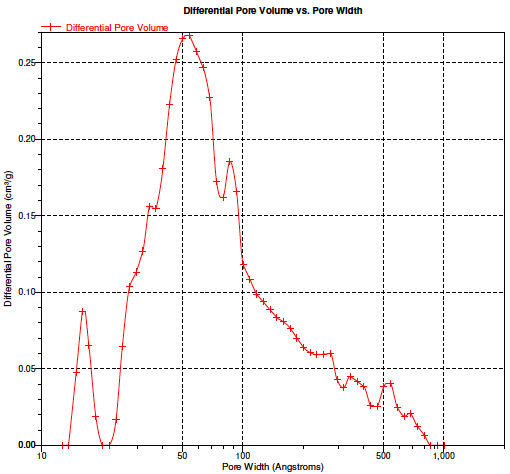
**

**Figure S10.** Pore size distribution of the RH_P_-Si-NH_2_-Pd(0) using the adsorption isotherms with density functional theory


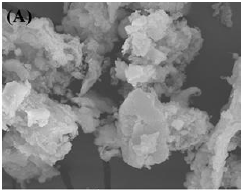


**Figure S11.** The SEM image of the RH-Si

1. Premi, C.; Jain, N. *Eur. J. Org. Chem.* **2013**, 5493−5499. [↑](#footnote-ref-2)
2. Thapa, S.; Gurung, S. K.; Dickie, D. A.; Giri, R. *Angew. Chem.* **2014**, *126*, 11804−11808. [↑](#footnote-ref-3)
3. Kanagaraj, K.; Pitchumani, K. *Chem. Eur. J.* **2013**, *19*, 14425−14431. [↑](#footnote-ref-4)
4. Tanaka, S.; Mori, A. *Eur. J. Org. Chem.* **2014**, 1167−1171. [↑](#footnote-ref-5)
5. Saikia, B.; Boruah, P. R.; Ali, A. A.; Sarma, D. *RSC Adv.* **2015**, *5*, 50655−50659. [↑](#footnote-ref-6)
6. Direct arylation of pyridines without the use of a transition metal catalyst. *Chem. Commun.* **2014**, 7124−7127. [↑](#footnote-ref-7)
